# A sticky situation – simple method for rapid poissonian encapsulation of highly aggregation-prone microbeads in polydisperse emulsions

**DOI:** 10.3389/fbioe.2025.1568027

**Published:** 2025-06-30

**Authors:** Filip Hasecke, Wolfgang Hoyer

**Affiliations:** Institut für Physikalische Biologie, Heinrich-Heine-Universität, Düsseldorf, Germany

**Keywords:** microbeads, in vitro compartmentalization, emulsion, encapsulation, aggregation, clustering, poisson distribution

## Abstract

Directed evolution leverages the principles of natural selection to engineer biomolecules with desired properties. Microbead-based approaches within water-in-oil emulsions have proven invaluable for high-throughput *in vitro* selections. However, highly aggregation-prone microbeads present significant challenges, including clustering, inconsistent distribution, and droplet instability. Here, we introduce a simple and cost-effective method for generating polydisperse emulsions with restored Poissonian distributions of highly aggregation-prone microbeads. This approach utilizes modified gel loader pipette tips, drawn out to create nozzles capable of disrupting microbead clusters during emulsification. Two widely utilized oil-surfactant formulations—mineral oil with Abil EM 90 and FluoSurf in HFE 7500 — were evaluated for emulsion preparation. Emulsions prepared using the modified nozzles exhibited exceptional stability, maintaining integrity during week-long incubations at 37°C, and reliably distributed microbeads into droplets in accordance with a Poissonian distribution despite the microbeads’ highly aggregation-prone property.

## 1 Introduction

Directed evolution is a powerful tool for engineering biomolecules with desired properties by mimicking the natural evolutionary process in a laboratory setting (Arnold and Volkov, 1999; Stucki et al., 2021; Wang et al., 2021). Among various *in vitro* methodologies, microbead-based approaches conducted in emulsions have gained significant attention for their ability to screen vast libraries of variants efficiently (Griffiths and Tawfik, 2003; Gan et al., 2008; Diamante et al., 2013; Price et al., 2014; Zhu et al., 2015; Mankowska et al., 2016; Siu et al., 2021; Anyaduba et al., 2022; Iizuka et al., 2022; Ito et al., 2024). These methods leverage the compartmentalization provided by water-in-oil emulsions to isolate individual genetic variants and their corresponding encoded proteins, ensuring genotype-phenotype linkage. Microbeads can be introduced to serve as a solid support for capturing and localizing genetic material or the expressed proteins, often through specific binding interactions (Diamante et al., 2013; Mankowska et al., 2016), or molecular probes to detect enzymatic activity (Griffiths and Tawfik, 2003; Zhu et al., 2015). The emulsion droplets act as microreactors, enabling high-throughput screening by creating millions of independent compartments, each containing a single microbead and the biochemical machinery for transcription and translation. The tight spatial control facilitates direct coupling between the genotype (DNA on the microbead) and the phenotype (a probe on the microbead detecting the functional activity of the protein).

There are two prominent approaches to prepare these emulsions: Highly controlled droplet-based microfluidics (Price et al., 2014; Song et al., 2003; Agresti et al., 2010; Goto et al., 2020) and preparation via stochastic one-step polydisperse emulsification methods (Gan et al., 2008; Mankowska et al., 2016; Siu et al., 2021; Tawfik and Griffiths, 1998; Griffiths and Tawfik, 2006; Wang et al., 2014; Byrnes et al., 2018).

Microfluidics-based methods utilize precisely engineered chips to manipulate small volumes of fluids at the nano-to microscale, enabling the creation of highly monodisperse droplets. Microfluidics chips, often made of PDMS (Song et al., 2003; Raj and Chakraborty, 2020) or glass (Aralekallu et al., 2023), feature intricate microchannel networks designed for controlled droplet generation and manipulation (Song et al., 2003).

Stochastic one-step polydisperse emulsification methods are used to prepare bulk emulsions using simple vortexing or mixing techniques, requiring minimal specialized equipment (Tawfik and Griffiths, 1998).

Each approach offers distinct advantages and disadvantages. Microfluidics-based systems offer precise control allowing for the generation of monodisperse droplets with highly uniform sizes, ensuring consistent reaction conditions across all compartments. On-chip manipulations of droplets such as mixing (Song et al., 2003), splitting (Link et al., 2004), merging (Song et al., 2003; Link et al., 2006), incubation (Song et al., 2003) and sorting via fluorescence or absorbance readouts (Agresti et al., 2010; Link et al., 2006; Ahn et al., 2006; Holstein et al., 2021), or droplet load and size (Nam et al., 2012; Jing et al., 2015) enable complex experimental workflows. However, microfluidics-based systems require special equipment and there are technical limits to throughput. As droplets are generated and processed in succession, the experiments are limited by flow speeds which leads to large-scale screening experiments consuming entire days of hands-on work and unfortunately needing to be closely monitored during the run, as they are highly sensitive to flow fluctuations and prone to clogging by particulate matter.

Stochastic one-step droplet generation can be performed in a single batch via mixing or vortexing of the oil- and aqueous-phases (Gan et al., 2008; Mankowska et al., 2016; Siu et al., 2021; Tawfik and Griffiths, 1998; Griffiths and Tawfik, 2006; Wang et al., 2014; Byrnes et al., 2018). This can be done in virtually any laboratory and does not require any specialized equipment. Another advantage is scalability, as larger volumes of emulsion can be generated with ease and can handle substantial library sizes without the constraints of microfluidic device throughput (Gantz et al., 2023). Furthermore, these approaches are most cost-effective, as they are inexpensive to set up and execute and consume less reagent than microfluidics-based systems. However, droplets in bulk emulsions are polydisperse. They vary in size, leading to inconsistent reaction conditions and potential bias in selection outcomes. Furthermore, the risk of cross-contamination between droplets is higher, which can compromise the fidelity of *in vitro* evolution processes.

The incorporation of microbeads into microfluidic systems presents significant technical challenges (Chen et al., 2023a). For successful addition of microbeads into droplets using microfluidic chips, it is crucial to ensure a uniform dispersion and dilution of microbeads in the precursor solution prior to droplet formation (Anyaduba et al., 2022). The efficiency of these approaches is usually constrainted by the Poisson distribution. Microbead encapsulation occurs randomly, with a theoretical maximum of only 37% of droplets containing a single microbead. This inefficiency results in significant waste of both microbeads in droplets containing more than one microbead and reagent in empty droplets. Recently, microfluidic strategies emerged that have overcome the limitation imposed by random Poisson distribution during microbead encapsulation into droplets (Chen et al., 2023a; Abate et al., 2009; Link et al., 2022; Yue et al., 2022; Luo and Lee, 2023). These approaches mostly rely on close packing of microbeads enabling controlled release and precise single-microbead addition into droplets. However, these approaches require the microbeads to be compressible and lubricous to prevent clogging (Chen et al., 2023a; Abate et al., 2009; Yue et al., 2022; Luo and Lee, 2023). Another approach uses surface acoustic waves (SAW) to actively encapsulate single cells into droplets. This method uses laser-assisted detection of individual cells during droplet formation and actively cutting-off droplets at the T-junction upon detection of a cell. This approach, however, results in the formation of polydisperse emulsions and requires diluted and nicely dispersed particles (Link et al., 2022).

In any of these cases, particle aggregation represents the nemesis of these microfluidics-based particle encapsulation approaches. Clumping of microbeads must be avoided, as their settling in the feed line can result in uneven encapsulation, disruption of droplet formation, or complete clogging of microfluidic channels. Certain microbead surface modifications—which might be necessary for your experimental setup—can exacerbate microbead aggregation, making it extremely difficult, if not impossible, for microfluidic systems to handle these samples effectively. When designing a selection scheme for a directed evolution campaign, predicting the effects of specific surface modifications on microbead aggregation remains challenging. Hydrophobic surface modifications, such as those introduced by certain peptides, proteins, fluorescent dyes, or polycyclic aromatic hydrocarbons, can be expected to promote microbead aggregation (Bharti et al., 2011). But also the conjugation of nucleic acids, particularly single-stranded DNA or RNA, can enhance aggregation due to complementary strand hybridization (Valignat et al., 2005; Leslie et al., 2012; Sloan et al., 2016). The exact influence of individual surface chemistries is often difficult to predict, and aggregation may unexpectedly occur and jeopardize the experimental setup. And in other cases, surface modifications that are expected to induce microbead aggregation may be unavoidable due to experimental requirements.

In such cases, polydisperse emulsification methods offer a viable alternative. These methods are less susceptible to issues related to microbead settling because precursor solutions are typically processed immediately, eliminating the idle time required in microfluidic systems. However, even polydisperse emulsification techniques face limitations when dealing with highly aggregation-prone microbeads. In case microbead clusters survive sonification and other means of dispersion or rapidly reassemble, clumps of multiple microbeads may still be encapsulated into single droplets.

In this study, we present a simple and effective method for rapid generation of polydisperse emulsions containing a restored Poisson distribution of highly aggregation-prone microbeads. Our approach utilizes drawn-out 200 μ l gel-loading pipette tips that act as thin nozzles. By forcing the microbead dispersion through the narrow nozzle orifice during encapsulation, microbead clusters are physically disrupted, ensuring the compartmentalization of isolated microbeads enabling the recovery of Poissonian distribution.

## 2 Results and discussion

**FIGURE 1:**
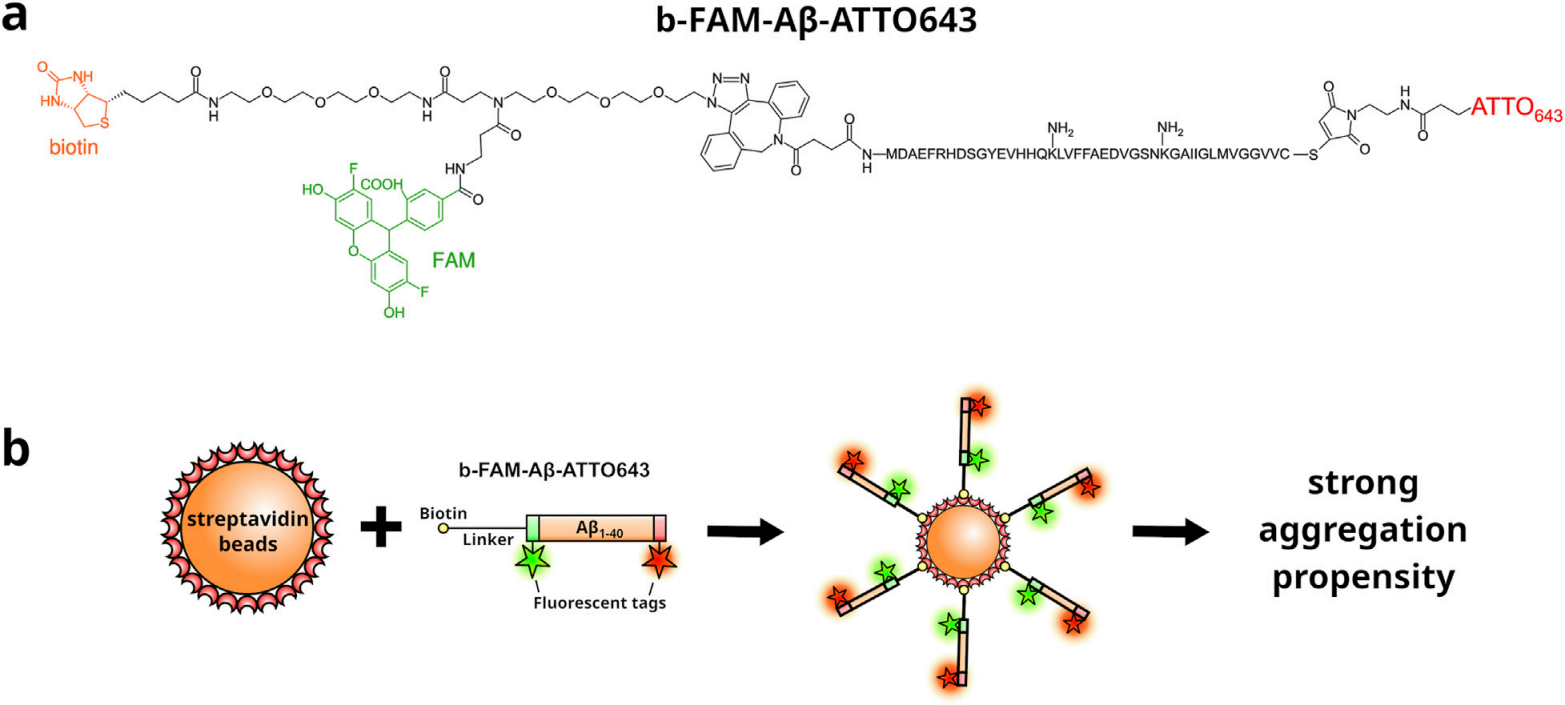
Schematic representation of the b-FAM-A β -ATTO643 peptide and the microbead functionalization approach. **(a)** Chemical structure of the b-FAM-A β -ATTO643 peptide. The peptide includes a biotin moiety for microbead attachment and a 5′ FAM fluorescent dye linked via a PEG spacer to the N-terminus, and an ATTO643 dye at the C-terminus. **(b)** Diagram of the microbead functionalization process, showing streptavidin-coated microbeads conjugated with the b-FAM-A β -ATTO643 peptide, which results in exacerbated aggregation behavior.

As part of an ongoing project, we required the encapsulation of individual microbeads functionalized with a specifically engineered peptide into droplets. This effort was integral to a directed evolution strategy aimed at developing proteolytically active antibodies targeting the amyloid- β (A β ) peptide. Our specifically designed peptide consisted of a highly modified A β _1–40_ peptide, which included a biotin moiety and a 5′FAM fluorescent dye, connected via a PEG linker to its N-terminus, along with an ATTO643 dye conjugated to its C-terminus (henceforth this peptide is referred to as b-FAM-A β -ATTO643, see Figure 1a). While the A β peptide is already inherently prone to aggregation, our modifications further exacerbated this tendency. Consequently, functionalizing streptavidin-decorated microbeads (ProMag 3 HP, Bangs Laboratories) with this peptide led to persistent and problematic cluster formation among the microbeads (see Figures 1b, 3a). Here, we aimed to develop a simple and reliable method to encapsulate these microbeads into droplets, addressing the challenges posed by their aggregation propensity and achieving a restored Poissonian distribution within droplets. We hypothesized this would be possible by forcing the microbead suspension through a tiny nozzle, which would physically disrupt microbead clusters during emulsion preparation allowing for Poissonian distribution of individual microbeads into droplets.

### 2.1 Homemade pipette tip-based nozzle

**FIGURE 2:**
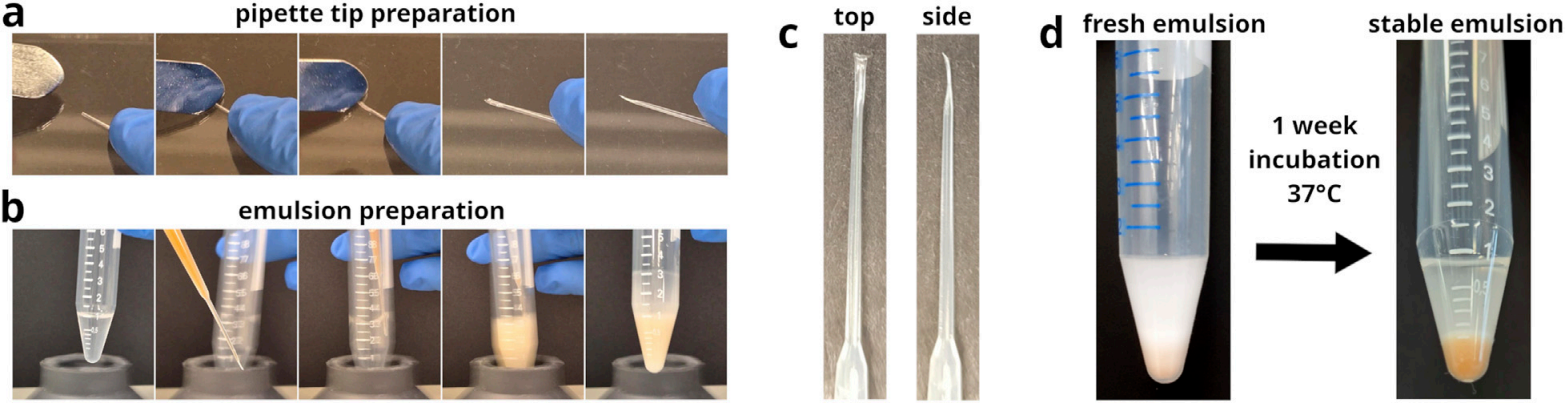
Preparation of modified gel loader pipette tips and their application as nozzles in emulsion generation. **(a)** Image series of the process used to modify 200 μ L gel loader pipette tips. The tip is compressed and elongated manually using the back of a scalpel handle, creating a drawn-out nozzle. **(b)** Image series illustrating the emulsification setup using a modified gel loader pipette tip, where the aqueous phase is gradually added to the oil-surfactant blend during vortexing. **(c)** Showcase of the modified pipette tip nozzle with its elongated and flattened nozzle design. **(d)** Stability test of an emulsion prepared using the modified pipette tips. Emulsions appeared intact after 1 week of incubation at 37°C, with no signs of broken droplets and no visible coalescence (see also Supplementary Figure S6) of any released microbeads at the bottom of the tube.

Low-cost and easily fabricated nozzles were created by modifying 200 μ l gel loader pipette tips (Sarstedt). This was achieved by manually compressing the frontmost 2 mm of the pipette tips using the handle side of a metal scalpel handle. The scalpel handle was gently drawn across the tip under moderate pressure (approx. 1 kg), elongating and flattening the nozzle (see Figure 2a, c and Supplementary Video S1). A single pass with the scalpel handle was typically sufficient to deform the pipette tip adequately. The patency of the modified pipette tips was assessed by aspirating 200 μ l microbead suspension using a 200 μ L pipette. Proper functionality was confirmed when the flow of the microbead suspension into the pipette tip was restricted, allowing complete aspiration within 5–10 s following rapid full extension of the pipette piston (see Supplementary Video S2). If liquid flow was not sufficiently restricted, allowing complete aspiration in less than 5 s, pipette tip nozzles could sometimes be rescued with a careful second pass with the scalpel handle. Pipette tip-derived nozzles that required longer aspiration times or were entirely obstructed were discarded.

The morphology of the pipette tip-derived nozzles can be quite diverse (see Supplementary Figures S1, S2). However, adequate flow restriction was the important factor to achieve sufficient microbead dispersion during emulsification (see Supplementary Figure S3). Differences in nozzle morphology did not noticeably affect their functionality.

### 2.2 Preparation of emulsions containing highly aggregation-prone microbeads

Emulsions were prepared using two widely utilized oil-surfactant formulations for comparison: the first consisted of mineral oil with 2% (v/v) Abil EM 90% and 0.05% (v/v) Triton X-100 (Anyaduba et al., 2022; Miller et al., 2006; Courtois et al., 2008; Tubeleviciute and Skirgaila, 2010; C Hatch et al., 2011; Turchaninova et al., 2013; Takeuchi et al., 2014; Tanaka et al., 2015; Zhang et al., 2020; Tanno et al., 2020; An et al., 2020), while the second employed a commercially available formulation, 2% FluoSurf (Dolomite), comprised of the inert perfluorocarbon carrier oil HFE 7500 and a proprietary fluorous surfactant (Bussiere et al., 2019; Karamitr et al., 2020; Zhu et al., 2023).

The aqueous phase consisted of PURExpress *in vitro* Protein Synthesis reagent (New England Biolabs) containing 10^7^ microbeads decorated with 10^6^ molecules b-FAM-A β -ATTO643 per microbead. PURExpress *in vitro* Protein Synthesis reagent is widely used in directed evolution experiments (Diamante et al., 2013; Mankowska et al., 2016; Iizuka et al., 2022; Goto et al., 2020; Holstein et al., 2021; Takeuchi et al., 2014; Diefenbach et al., 2018; Lindenburg et al., 2020; Lindenburg and Hollfelder, 2021; Zeng et al., 2023). However, both microbead surface-modifications (Valignat et al., 2005; Leslie et al., 2012; Sloan et al., 2016; Castro et al., 2016; Arya et al., 2017) and the use of *in vitro* protein synthesis reagent can increase the aggregation propensity of microbeads (see Supplementary Figure S4).

**FIGURE 3:**
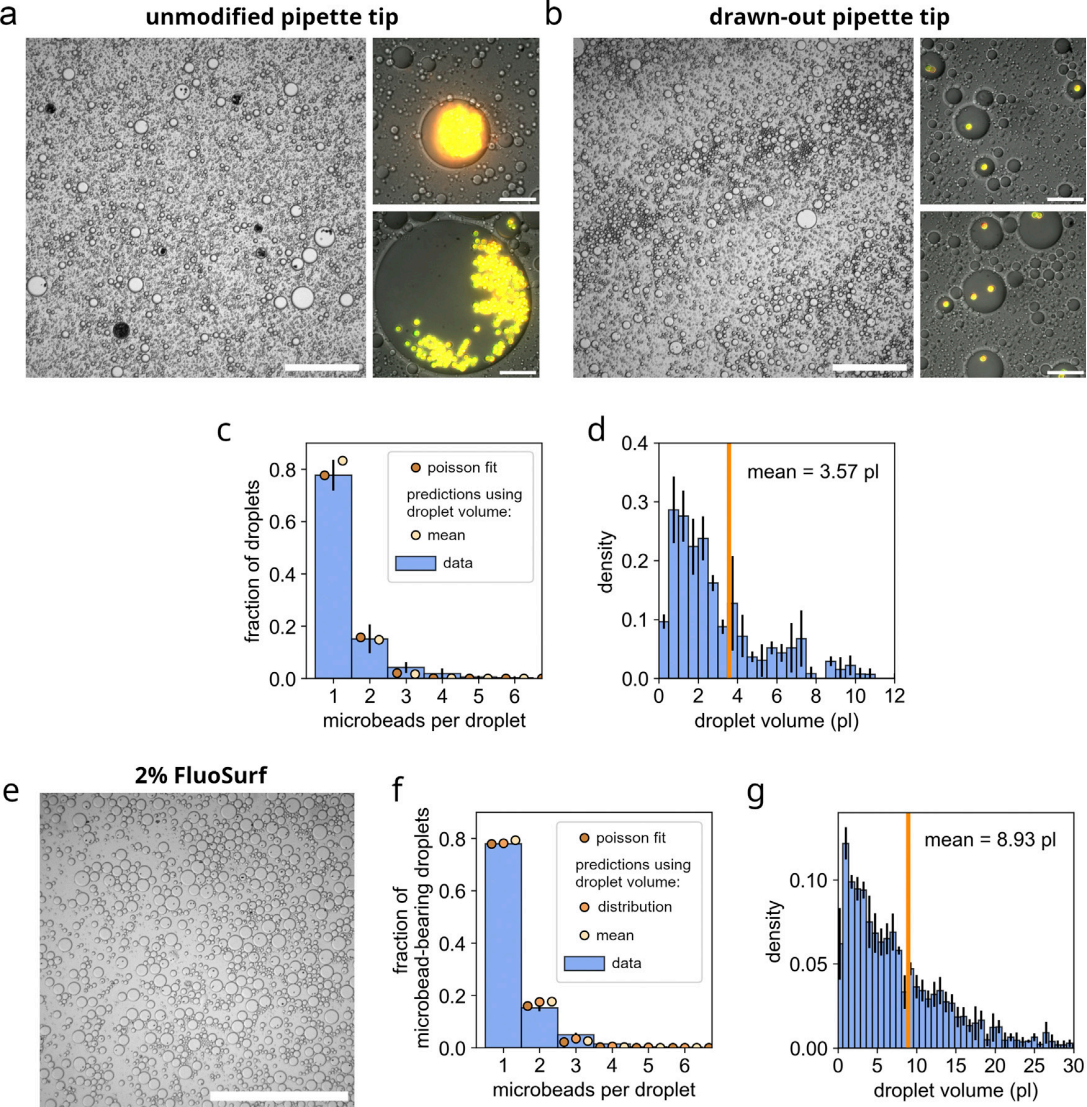
Restoration of Poissonian distributions of microbeads in emulsion droplets using gel loader pipette tip-derived nozzles. **(a)** Microscopic image of a control emulsion (Abil EM 90-based) prepared with unmodified gel loader pipette tips, showing severe clustering of microbeads and a low fraction of droplets containing microbeads. The overview image is captured using DIC microscopy at low magnification (scale bar: 250 μ m). Zoomed insets display DIC images overlaid with fluorescence (488 and 633 nm excitation, scale bar: 25 μ m) **(b)** Microscopic image of an Abil EM 90-based emulsion generated with a modified gel loader pipette tip-derived nozzle, showing a restored Poissonian distribution of microbeads within droplets. Overview DIC image, scale bar 250 μ m. Zoomed insets, DIC and fluorescence overlays (488 and 633 nm excitation, scale bar: 25 μ m). **(c)** Quantitative analysis of microbead encapsulation in Abil EM 90-based emulsion droplets generated using modified gel loader pipette tip-derived nozzles. The fraction of droplets containing n microbeads follows a Poissonian distribution. Error bars represent standard deviation. N = 4 emulsions were prepared with a total of 360 microbead-containing droplets analyzed. Microbead distribution aligned with a Poissonian distribution fit (brown dots). Expected Poisson distributions of microbeads were predicted based on the mean microbead-contianing droplet volume and the total aqueous reagent volume (beige dots). **(d)** Droplet volume (pL) histogram of microbead-containing droplets in Abil EM 90-based emulsions prepared with modified nozzles. The mean volume is 3.6 pL (orange vertical line). Error bars indicate standard deviation. **(e)** Microscopic image of a FluoSurf-based emulsion prepared with a gel loader pipette tip-derived nozzle, demonstrating restored Poissonian microbead distributions within droplets. DIC image captured at low magnification (scale bar: 500 μ m). **(f)** Quantitative analysis of microbead encapsulation in droplets of FluoSurf-based emulsions generated using the modified gel loader pipette tip nozzles. Shown is the fraction of microbead-bearing droplets containing n amount of microbeads. Error bars denote the standard deviation. N = 3 emulsions were prepared and a total of 1.963 microbead-containing droplets were analyzed. Microbead distribution aligned with a Poissonian distribution fit (brown dots). Expected Poisson distributions of microbeads were predicted based on the mean microbead-contianing droplet volume and the total aqueous reagent volume (beige dots) and based on the droplet volume distribution (orange dots). **(g)** Histogram of microbead-containing droplet volumes (pL) for FluoSurf-based emulsions prepared with modified pipette tips. The mean volume is indicated by the orange vertical line (8.9 pL). Error bars denote the standard deviation.

As a control, an Abil EM 90-based emulsion was prepared using an unmodified gel loader pipette tip. 100 μ l aqueous phase was gradually (over the period of 10 s) added to 1 mL of the oil-surfactant blend while vortexing at a low speed (setting 2–3 of 6 on a VV3 vortexer by VWR, which corresponded to 1200 RPM). The procedure is illustrated in Figure 2b and Supplementary Video S3. For the creation of Abil EM 90-based emulsions, maintaining a low vortex speed is essential to prevent air entrapment, which can compromise emulsion stability. In the resulting control emulsion, the microbeads exhibited severe clustering, with only a small fraction of droplets containing any microbeads and those that did, contained hundreds of clustered microbeads (see Figure 3a). Additionally, the droplets were highly unstable, undergoing coalescence and rupture within minutes to hours. Over the course of minutes, free microbeads were found rapidly settling at the bottom of the tube, further indicating the instability of the emulsion (see Supplementary Figure S5).

In comparison, emulsions with the same composition were prepared using the modified, drawn-out gel loader pipette tips, as shown in Figures 2a–c. The resulting emulsions demonstrated exceptional stability, remaining intact during a 1 week incubation at 37°C without any signs of degradation (see Figure 2d; Supplementary Figure S6). Microscopic analysis revealed that microbead-containing droplets in Abil EM 90-based emulsions presented a mean volume of 3.6 pL (see Figure 3d). Wherein, approximately 80% contained a single microbead, less than 20% contained two microbeads and less than 5% contained three or more microbeads (see Figures 3b,c). Quantitative analysis of the microbead distribution within droplets closely followed a Poisson distribution fit (see Figure 3c, brown dots), yielding a fitted theoretical rate of occurence μ -value of 0.41 with a sum of squared errors (SSE) of 0.032. For comparison, the mean droplet volume of the analyzed microbead-containing droplets and the total aqueous reagent volume was used to approximate the expected rate of occurence, resulting in a μ -value of 0.36 (SSE: 0.045). The Poisson distribution derived using μ = 0.36 slightly deviated from the experimental data. Our data undercut the expected proportion of single-microbead droplets while the fraction of multi-microbead droplets exceeded expected occurence from the Poisson distribution (see Figure 3c, beige dots). However, this approximation did not account for the polydispersity of the emulsion droplets, as it was based solely on the mean droplet volume of microbead-containing droplets. The presence of larger droplets is expected to increase the frequency of multi-microbead droplets while decreasing the proportion of single-microbead droplets. Accurately capturing this effect would require an analysis of the overall droplet volume distribution. However, such an analysis was unfeasible for Abil EM 90-derived emulsions due to the limited resolution of the low-magnification overview microscopic images and the insufficient accuracy of automated image detection methods applied to analyze high-magnification images. In contrast to Abil EM 90-based emulsions, however, this analysis was feasible for FluoSurf-based emulsions, due to the overall larger droplet volumes and less prominent droplet coalescence when applied onto microscopic slides.

A similar investigation, as for the Abil EM 90-derived emulsions, was performed using emulsions prepared with a 2% FluoSurf oil-surfactant blend in perfluorocarbon carrier oil HFE 7500. Therefore, 200 μ l PURExpress protein synthesis reagent with 10^7^ microbeads was gradually (over the period of 10s) added to 600 μ l 2% FluoSurf blend while vortexing at high speed, carefully avoiding overspills (setting 5 of 6 on a VV3 vortexer by VWR, which corresponded to 2750 RPM). Following the addition of the aqueous phase, the emulsion was vortexed for an additional 5 minutes to ensure completion. The extended vortexing time was necessary to further reduce the mean droplet size and narrow down the size distribution. The resulting emulsions exhibited similar stability as the Abil EM 90-based emulsions but consisted of larger droplets, with a mean volume of 8.9 pL for microbead-containing droplets (see Figures 3e,g). Quantitative analysis of the microbead distributions within droplets again showed excellent alignment with a Poissonian distribution fit ( μ -value = 0.43, SSE = 0.0044) (see Figure 3f brown dots). In a similar fashion to the analysis of the Abil EM 90-derived emulsions, the expected true rate of occurence μ -value was derived from the mean microbead-containing droplet volume and the total aqueous reagent volume to calculated the corresponding expected Poisson distribution of microbeads within droplets ( μ -value = 0.45, SSE = 0.0060) (see Figure 3f beige dots), which was closely in line with the fitted Poisson distribution. Both analyses, however, underestimated the amount of droplets containing multiple microbeads compared to experimental data. This was assumed to have been due to the fact that both analyses ignored the polydispersity of the emulsion droplets and relied solely on the assumption of a uniform mean droplet volume. However, in a distribution, the presence of larger droplets would lead to a higher occurrence of multi-microbead droplets while reducing the proportion of single-microbead droplets, whereas smaller droplets would have the opposite effect. A precise representation of this effect would require an analysis of the overall droplet volume distribution. To approximate this effect, we analyzed the droplet volume distribution of FluoSurf-derived emulsions, including empty droplets. The distribution was divided into 2 pL size bins, and Poisson distributions were calculated for each fraction based on their respective share of the total aqueous reagent volume (see Supplementary Figure S7). A weighted sum of these distributions was used to estimate the expected microbead distribution across the entire polydisperse emulsion (see Figure 3f, orange dots), yielding the closest match to the experimental data (SSE 0.0041).

These results demonstrate that this simple and rapid technique, utilizing homemade nozzles created by drawing out gel loader pipette tips, reliably produces emulsions with restored Poissonian distributions of microbeads, even for highly aggregation-prone microbeads.

Further standardization of nozzle fabrication may be feasible using the protocol published by Chen et al. (2023b), which uses a Do-It-Yourself (DIY) screwdriver-based tip modification tool to deform pipette tip orifices into an eliptical shape. To adapt this protocol for our purpose, considerably higher torques would be required to achieve sufficient flattening of the pipette tips. An adaptation of this technique would allow for standardization of nozzle production and lead to further improvement in reproducibility. On a note, however, in our experiments ultimate droplet size distributions appeared to be primarily influenced by factors such as surfactant concentrations, vortex speeds, and vortex timings, with little impact from the actual nozzle morphology.

In conclusion, we have developed a simple, cost-effective, and efficient method for the rapid generation of polydisperse emulsions, enabling the encapsulation of highly aggregation-prone microbeads in accordance with a Poissonian distribution using modified gel-loading pipette tips. This low-cost approach requires minimal equipment and materials, making it highly accessible to laboratories with limited resources. By restoring the Poissonian distribution of aggregation-prone microbeads, this method addresses a significant challenge and expands the range of applications in directed evolution systems. Microbead modifications that typically induce aggregation no longer pose a barrier to establishing directed evolution campaigns. The method’s affordability, reproducibility, and adaptability make it a compelling solution for diverse bead-based assays and droplet-based applications that necessitate the incorporation of microbeads into droplets. Beyond microbeads, this technique is likely applicable to other aggregation-prone particulate matter, provided the particle size remains within the dimensions of the nozzle’s orifice. Furthermore, modulating the applied pressure to reduce compression of the pipette tips may further accommodate the use of larger particles to some degree. In addition, our technique is expected to be compatible with microorganisms possessing robust exterior shells, such as bacteria or yeast. However, its applicability to eukaryotic cells, including animal or human cells, is likely limited due to their susceptibility to damage from the excessive shear forces generated within the pipette tip-derived nozzles.

## 3 Materials and methods

### 3.1 Materials

200 μ l gel loader pipette tips were ordered from Sarstedt (Ref: 70.1190.100). Surfactants Abil EM 90 (Lot: E517C06179, product code: 201109) and 2% FluoSurf (S/N: FL010290, product number: 3200808) were from Evonik and Dolomite, respectively. Triton X-100 (Sigma-Aldrich, Lot: 053K00262V, catalog number: T9284). Light mineral oil (Sigma-Aldrich, Lot: MKCQ7240, catalog number: 330779). Peptide conjugation chemicals: Fluorescein-biotin-azide (catalog number: BP-28107) and Dibenzocyclooctin N-hydroxysuccinimide ester conjugate (DBCO-NHS, catalog number: BP-22231) were ordered from Click Chemistry Tools. ATTO643-maleimide (catalog number: AD 643-41) from ATTO-TEC. ProMag HP 3 Streptavidin microbeads (Lot: 15546, catalog number: PMS3HP) were ordered from Polysciences (distributor for Bangs Laboratories, Inc.). The *in vitro* transcription and translation reagent, PURExpress *In Vitro* Protein Synthesis Kit (catalog number: E6800L) was ordered from New England Biolabs.

### 3.2 Synthesis of b-FAM-A β -ATTO643

A precursor A β variant, MA β 40C, which contains an N-terminal methionine and a C-terminal cysteine residue, was recombinantly produced by coexpression of MA β 40C and the A β -binding Affibody ZA β 3, as previously described for the production of A β and dimA β (Macao et al., 2008; Hasecke et al., 2018). Both MA β 40C and ZA β 3 were encoded on the bacterial expression vector pACYCDuet-1 (Novagen) which was designed for bicistronic expression. MA β 40C was inserted into the first multiple cloning site (MCS) and ZA β 3, as a His-tagged (His) 6-ZA β 3 variant, was cloned into the second MCS of the vector. BL21 (DE3) E.coli cells were transformed with the plasmid and overnight precultures were prepared in 50 mL LB medium with 100 μ g/mL carbenicillin. The next day, 2 L LB medium with 100 μ g/mL carbenicillin were inoculated with 40 mL of the preculture. Cells were grown to an O.D. of 0.6 for approximately 3 h at 37°C and subsequently expression was induced by adding 1 mM isopropyl β -D-1-thiogalactopyranoside (IPTG) followed by further incubation at 37°C for 4 h. Cells were harvested by centrifugation and stored at −20°C. For purification of MA β 40C, cell pellets were resuspended in 50 mM sodium phosphate, 300 mM NaCl, 20 mM imidazole pH 8 supplemented with EDTA-free protease inhibitor tablets (Roche), as recommended by the manufacturer. Cells were lysed via cell disruptor (Constant Systems LTD) at 2.9 kbar and cell debris was removed via centrifugation at 31,026 × g (18,000 RPM in a Backman Coulter JA-20.1 rotor) at 4°C for 40 min. The MA β 40C:ZA β 3 complex was captured by immobilized metal affinity chromatography (IMAC) on a HisTrap 5 mL excel column (Cytiva) and MA β 40C was eluted via denaturation of the complex with 8 M Urea in 20 mM sodium phosphate pH 7. To remove residual ZA β 3, the eluate was first reduced by supplementing 5 mM TCEP and subsequently separated via RP-HPLC on a semi-preparative Zorbax 300SB-C8 RP-HPLC column (9.4 mm × 250 mm) connected to an Agilent 1260 Infinity system with UV detection at 214 nm and 275 nm. Monomeric MA β 40 C was eluted on a gradient of 12.5%–45% acetonitrile in water with 0.1% (v/v) trifluoroacetic acid over 15 min at 80°C. The eluate was lyophilized and powdered MA β 40C was stored at RT.

MA β 40C-ATTO643 was synthesized via maleimide conjugation to the C-terminal cysteine residue via succinimidyl thioether formation. 1 mg maleimide-ATTO643 was dissolved in 100 μ l DMF (final concentration: 9.33 mM) and 1 mg MA β 40 C was dissolved in 600 μ l 8 M Urea 20 mM sodium phosphate pH 7.0 supplemented with 5 mM TCEP (final MA β 40C concentration: 365 μ M). 33 μ l maleimide-ATTO643 solution was added to the MA β 40 C solution (1.4-fold excess of the dye) and incubated for 4 h at room temperature. The MA β 40C-ATTO643 product was purified via RP-HPLC by elution on a gradient of 25%–45% acetonitrile in water with 0.1% (v/v) trifluoroacetic acid over 15 min at 80°C while detecting the absorption at 643 nm. The MA β 40C-ATTO643 product peak was collected and lyophillized.

20 nmol MA β 40C-ATTO643 was dissolved in 500 μ l 50% Dimethylformamide (DMF) in 50 mM sodium phosphate pH 6.5. To the MA β 40C-ATTO643 solution 1.6 μ l 62.5 mM NHS-DBCO (25 mg in 1 mL DMF), for a 5-fold molar excess, and 26 μ l 9 mM biotin-FAM-azide (1 mg in 100 μ l DMF), for a 10-fold molar excess, was added and incubated overnight at 4°C on a tube roller. The reaction was carried out at a slightly acidic pH of 6.5 to promote preferential conjugation at the N-terminal primary amine instead of the primary lysine amines. Final purification was per formed via RP-HPLC. Final b-FAM-A β -ATTO643 product was eluted on a gradient of 25%–45% acetonitrile in water with 0.1% (v/v) trifluoroacetic acid over 15 min at 80°C while detecting the absorption at 488 nm and 643 nm. Product was lyophillized and aliquots were prepared by redissolving in HFIP followed by lyophilization in appropriate aliquot sizes. Subsequently, the products were stored at −80°C. Final product concentration was determined by dissolving one aliquot in PBS and subsequent UV-Vis spectroscopy. The absorption at 643 nm was measured to calculate the concentration with ϵ _643 nm_ = 150,000 M^−1^cm^−1^.

### 3.3 Preparation of functionalized microbeads

For functionalization of microbeads, 5 × 10^7^ ProMag 3 HP streptavidin microbeads were used. Microbeads were washed three times with 200 μ l bind and wash buffer (20 mM Tris, 1 M NaCl, 1 mM EDTA, 0.05% Triton X-100, pH 7.5) using a magnetic separator and finally resuspended in 100 μ l bind and wash buffer. A volume of 9.2 μ L of 9 μ M b-FAM-A β -ATTO643 in 1X PBS was added to the microbeads and incubated overnight at 7°C with shaking at 1,400 rpm in a thermomixer. Based on the total number of molecules calculated using Avogadro’s constant and assuming complete (lossless) binding of all peptide molecules to the microbeads, this corresponded to approximately 10^6^ peptides per microbead. Afterwards, the microbeads were washed three times with 200 μ l breaking buffer (10 mM Tris, 100 mM NaCl, 1% Triton X-100, 1% SDS, pH 8.0) and then incubated overnight at 1400 RPM 7°C in a thermomixer. Microbeads were washed three times with 200 μ l bind and wash buffer and subsequently resuspended in 100 μ l bind and wash buffer supplemented with 0.1% sodium azide (NaN_3_) and stored at 4°C until use.

### 3.4 Preparation of gel loader pipette tip-derived nozzle

Detailed step-by-step instructions are provided in the Supplementary Material.

Modified pipette tip nozzles were prepared by manually drawing out standard 200 μ L gel-loading pipette tips using the blunt handle end of a metal scalpel handle. All steps were conducted under aseptic conditions within a laminar flow hood. To ensure sterility, the scalpel handle was autoclaved prior to use.

Step-by-Step Instructions:

1. A gel loader pipette tip was positioned on the flat surface of a sterile 96-well plate lid (Nunc, catalog number: 243656).
2. The handle side of a metal scalpel was pressed onto the frontmost 2 mm of the pipette tip, with the handle tilted at a 45°angle toward the pipette tip orifice.
3. The scalpel handle was drawn across the pipette tip toward the orifice while applying approximately 1 kg of pressure, as determined by a precision scale. A single pass was typically sufficient; however, if compression was incomplete, a second pass could be performed to ensure proper modification.

The preparation process is demonstrated in Supplementary Video S1, and a gallery of different nozzle preparations and their QC properties is provided in to 3 Supplementary Figure S1.

#### 3.4.1 Quality control

The proper fabrication of pipette tip-derived nozzles was usually evaluated by aspirating 200 μ L of a suspension of 10^7^ microbeads in PURExpress protein synthesis reagent using a 200 μ L pipette. We performed QC aspiration tests immediately before emulsification generation with the same microbead/reagent mixture that was used for the emulsification experiment. However, comparison of liquid compositions on aspiration times revealed no significant difference between aspiration of 200 μ l MQ water, 200 μ l MQ water with 10^7^ microbeads and 200 μ l PURExpress reagent with 10^7^ microbeads (see Supplementary Figure S3) and therefore QC can also be done with water alone. QC was performed by aspiration via rapid, full extension of the pipette piston. With properly prepared nozzles the liquid should be aspirated gradually over a period of 5–10 s. If aspiration was completed in less than 5 s, indicating insufficient flow restriction, a second pass with the scalpel handle could occasionally rectify the issue. Nozzles exhibiting excessively slow aspiration or complete blockage were discarded.

The quality control procedure is demonstrated in Supplementary Video S2 and Supplementary Figures S2, S3.

It was observed that variations in external nozzle morphology due to variation in the preparation did not noticeably influence the emulsions and dispersion of microbeads. Proper dispersion of microbeads was rather dependent on sufficient nozzle compression.

### 3.5 Emulsification

Detailed step-by-step instructions are provided in the Supplementary Material.

10^7^ microbeads decorated with 10^6^ molecules b-FAM-A β -ATTO643/microbead were used. Microbeads were washed twice with 100 μ l 1x PBS and twice with 100 μ l 1x PBS, 1 mg/mL UltraPure BSA (Invitrogen) using a magnetic rack. Afterwards, microbeads were separated from the liquid using the magnetic rack and the whole aqueous solution was removed. Microbeads were resuspended in 100 μ l or 200 μ l PURExpress Protein Synthesis reagent (prepared according to manufacturer’s instructions), for Abil EM 90-based and FluoSurf-based emulsions, respectively. The aqueous solution with dispersed microbeads was kept on ice and incubated for 10 min.

For Abil EM 90-based emulsions 1 mL mineral oil, 2% (v/v) Abil EM 90, 0.05% (v/v) Triton X-100 was used as oil-surfactant mixture. Oil-surfactant mixture was transferred to a conical 15 mL tube and vortexed, albeit at a low speed to prevent the introduction of air. The vortexer VV3 by VWR was used on the setting 2–3 of 6, which resulted in a 1200 RPM (as determined by high speed video footage) circular path motion with a 5 mm orbital diameter. The aqueous solution with dispersed microbeads was briefly sonicated for 5 s in a sonicator bath. The 100 μ l aqueous solution was aspirated into either a modified or unmodified 200 μ l gel loader pipette tip and then slowly transferred (over the course of 10 s) to the vortexing oil surfactant mixture. After addition, the emulsion was vortexed for 10 more seconds, finalizing the emulsion.

For FluoSurf-based emulsions, the aqueous phase was treated the exact same way, but then gradually added (over the course of 10 s) to 600 μ L 2% FluoSurf oil-surfactant blend while vortexing at a high speed on setting 5 of 6 (2750 RPM as determined by high speed video footage) on a VV3 vortexer by VWR. After addition of the aqueous phase the emulsion was finalized by vortexing for 5 more minutes. With the extended vortexing time, the droplet sizes were further reduced, and the size distribution became more narrow. This prolonged vortexing was not necessary for Abil EM 90-based emulsions, which formed a fine emulsion immediately upon addition of the aqueous phase.

The emulsification procedure can be viewed in Supplementary Video S3.

### 3.6 Microscopic analysis

Microscopic imaging of emulsions was conducted using a Leica Infinity TIRF microscope operated in epifluorescence and differential interference contrast (DIC) mode using Leica LAS AF software. An 8 μ L emulsion aliquot was placed onto a 60 × 24 mm cover slip and topped with a smaller 20 × 20 mm cover slip. Overview images were captured using a Leica HC PL FLUOTAR 10 × /0.30 DRY objective, while high-magnification images were acquired with a Leica HCX PL APO 100 × /1.47 OIL objective. DIC imaging was performed at both magnifications, and epifluorescence images at high magnification were recorded using excitation wavelengths of 488 nm with an emission band pass filter of 507–543 nm and excitation at 633 nm with an emission band pass filter of 669–741 nm.

### 3.7 Data analysis

Microscopic image analysis was conducted using FIJI software (ImageJ, version 1.54f). For Abil EM 90-based emulsions, high-magnification images were utilized, as accurate differentiation of microbeads and droplet volumes was not achievable from overview images. In contrast, FluoSurf-based emulsions were analyzed using lower-magnification overview images due to the larger droplet sizes, enabling the analysis of a larger number of droplets. Diameters of droplets harboring microbeads were measured using FIJI’s ruler tool and microbeads were counted. A total of N = 4 emulsions with a total of 360 microbead-containing droplets were analyzed for Abil EM 90-based emulsions, and a total of N = 3 emulsions with a total of 1.963 microbead-containing droplets were analyzed for FluoSurf-based emulsions. Furthermore, for FluoSurf-based emulsions the droplet volume distribution (including empty droplets) was determined based on 4.874 analyzed droplets from N = 3 emulsions.

Further data analysis was performed using Python (version 3.12.8). Numpy (version 2.1.3) and Pandas (version 2.2.3) were used for data storage and handling. Matplotlib (version 3.9.3) and Seaborn (version 0.13.2) were used to render Figures. Scipy (version 1.14.1) was used to fit Poisson distributions.

Droplet volumes were determined based on the measured droplet diameters and the liquid layer height between the microscopy slide and cover slip. Liquid layer heights for Abil EM 90-based and FluoSurf-based emulsions were measured by focussing on the top of the bottom glass slide (marked by scratches with a scalpel) and then focussing on the bottom of the top glass cover slip (also marked by scratches with a scalpel) and recording the traveled z-level distance. Liquid layer heights for Abil EM 90-based and FluoSurf-based emulsions were determined to be 8.4 μ m and 7.2 μ m, respectively. Volumes were calculated using the sphere volume formula for all droplets with a diameter less than the liquid layer height and for droplets with a diameter larger than the liquid layer height the volume formula for cylinders was used.

Poisson distributions were fitted using the probability mass function (pmf) of the scipy.stats.poisson library which utilizes the following Equation 1:

$$f\left(k\right)=exp\left(-\mu \right)\frac{\mu^{k}}{k!}$$ (1)

with k being the number of microbeads contained in a droplet and μ being the fitted expected rate of occurrences.

## Data Availability

The original contributions presented in the study are included in the article/Supplementary Material, further inquiries can be directed to the corresponding author.
