# Supplementary material for: A sticky situation – simple method for rapid poissonian encapsulation of highly aggregation-prone microbeads in polydisperse emulsions
Source: Front Bioeng Biotechnol. 2025 Jun 30;13:1568027. doi: 10.3389/fbioe.2025.1568027 (PMC12256949; doi:10.3389/fbioe.2025.1568027)
Supplement: Supplementary file 2 [file DataSheet1.pdf]

Supplementary Information for

A sticky situation – Simple method for rapid Poissonian encapsulation of highly aggregation-prone microbeads in polydisperse emulsions

Hasecke et al.

Step-by-step instructions for the preparation of pipette tip-derived nozzles

Preparations in case sterility is required:

- Autoclave a metal scalpel handle (without a knife) to ensure sterility.
    - e.g. scalpel handle No. 4 (Carl Roth, Art. No. 1PYN.1)
- Set up all materials inside a laminar flow hood to maintain aseptic conditions.
    - 200 µl gel loader pipette tips (Sarstedt, ref: 70.1190.100)
    - sterile 96-well plate lid (e.g. Nunc, catalog number: 243656).

Pipette tip modification procedure:

- Place 200 µL gel-loading pipette tip on the flat surface of a sterile 96-well plate lid
- Press the thin flexible catheter part of the pipette tip flat onto the surface with your index finger and secure stable positioning of the pipette tip with your hand (as can be seen in Supplementary Video 1 and main Figure 2 a)
- Place the sterilized scalpel handle with the rounded handle side on the frontmost 2 mm of the pipette tip at a 45° angle
- Apply approximately 1 kg of pressure using the scalpel handle (pressure verified via precision scale) and draw the scalpel handle across the pipette tip towards the orifice in a single, smooth motion (see Supplementary Video 1 and main Figure 2 a).
    - The applied pressure should be monitored using a precision scale while learning the technique, once reliably mastered the scale is usually not required
- Inspect the pipette tip by performing QC:
    - If the compression is adequate according to QC, proceed with emulsification experiment.
    - If incomplete, perform a second pass using the same method.

Quality Control (QC) procedure for pipette tip-derived nozzles

Prepare the QC test solution:

- Different solution compositions can be used as QC test solution without detrimental effect to the QC
  - If feasible, use the microbead + reagent mixture that shall be emulsified as QC test solution (200 µl required for QC). For example, 10^7 microbeads in 200 µl PURExpress protein synthesis reagent
  - Alternatively, prepare a solution of 10^7 microbeads in 200 µl water as QC solution
  - Otherwise, 200 µl pure water can be used as QC solution, however, this approach does not assess potential nozzle clogging caused by the presence of microbeads.

Perform QC:

- Apply the to be tested pipette tip-derived nozzle on a 200 µl pipette (we used an Eppendorf Research plus, 20 – 200 µL variable volume pipette)
- Setup a timer
- Simultaneously start the timer and aspirate the QC solution by rapidly and fully depressing the pipette piston
- Stop timer, when the entire 200 µl QC solution was aspirated

Evaluate QC results

- QC is **successful**, if:
  - QC solution was gradually aspirated over **5–10 seconds**
    - ➢ **Continue with emulsification experiment**
- Nozzle is **undercompressed**, if:
  - QC solution was aspirated in less than 5 seconds
    - ➢ Perform a **second compression pass** with the scalpel handle
- QC is **failed**, if:
  - Aspiration is too slow (**beyond 10 seconds**), or **complete blockage** occurs
    - ➢ **Reject nozzle**

Step-by-step instructions and considerations for the preparation of microbead-containing emulsions

Preparation of microbeads: All microbead modifications specific to the experimental setup should be completed prior to emulsification. We recommend using microbeads with a nominal diameter of 3 µm or larger, as they offer sufficient surface area for most downstream applications (e.g., fluorescence detection via fluorescence-activated cell sorting [FACS]) and are easier to handle. In contrast, microbeads with diameters of 1 µm or smaller are more susceptible to loss during recovery from emulsions and washing steps.

Immediately before emulsification, microbeads should be washed twice with 1× PBS and subsequently twice with 1× PBS containing 1 mg/mL UltraPure BSA (Invitrogen). The inclusion of BSA can in some cases help reduce microbead aggregation and block uncoated surfaces.

Considerations before emulsion preparation:

For emulsions based on Abil EM 90, we recommend an aqueous reaction volume of 100–200 µL per 10^7 microbeads. In contrast, FluoSurf-based emulsions typically require a minimum volume of 200 µL, due to the generally larger droplet size compared to those formed with Abil EM 90. Reducing the aqueous phase volume below these thresholds may lead to droplet overcrowding, resulting in multiple microbeads per droplet.

The choice of vortexing equipment affects the reproducibility of emulsion formation. We recommend using a vortex mixer that exhibits a concentric circular motion, such as the VV3 model from VWR. Devices that operate with a pear-shaped motion, such as the VWR model No. 444-1372, are less suitable due to inconsistent emulsification dynamics.

In principle, any aqueous solution can be used to prepare emulsions. In our step-by-step protocol, we illustrate emulsion preparation using the PURExpress in vitro protein synthesis system as the aqueous phase. However, other reagents — such as PCR master mixes for on-bead emulsion PCR or liquid culture media for microbial incubation — can be employed, depending on the intended application. In certain cases, the addition of 0.5% Triton X-100 to the aqueous phase may enhance emulsion stability.

Step-by-step instructions for the preparation of Abil EM 90-based emulsions

- Prepare mineral light oil surfactant mixture by mixing 960 µl mineral oil with 40 µl Abil EM 90 and 0.5 µl Triton X-100 for a final concentration of 2% (v/v) Abil EM 90, 0.05% (v/v) TritonX-100 in mineral light oil.
- Separate 10^7 microbeads from liquid using a magnetic rack for paramagnetic microbeads or via centrifugation for non-paramagnetic microbeads.
- Resuspend microbeads in 100 µl to 200 µl PURExpress Protein Synthesis reagent (prepared according to manufacturer's instructions).
- Keep microbead suspension on ice.

- Transfer 1 ml oil-surfactant mixture to a 15 ml conical tube.
- Vortex the oil-surfactant mixture without a lid. Use a low speed to prevent the introduction of air during emulsification. In our case, the vortexer VV3 by VWR was used on the setting 2–3 of 6 (1200 RPM) with a 5 mm orbital diameter.
- Sonicate the aqueous microbead suspension briefly for 5 seconds in a sonicator bath
- Aspirate the microbead suspension into a pipette tip-derived nozzle.
- Slowly (over the course of 10 s) add the microbead suspension to the vortexing oil surfactant mixture. Keep the pipette tip orifice close to the wall of the conical tube during the addition (see Supplementary Video 3).
- After addition, continue vortexing for **5-10 more seconds** to finalize the emulsion.
- Check the emulsion via microscopy. Transfer 8 µl of the emulsion on a microscopy slide and place a microscopy cover on top. Best practice: Evaluate the monoclonality of the microbeads in emulsion droplets. When overcrowding with microbeads is a persistent issue, increase the volume of the aqueous reagent or reduce the amount of microbeads. Furthermore, evaluate droplet diameters microscopically. Droplet diameters can be further reduced by increasing the additional vortex time after addition of the aqueous phase.
- The total emulsion volume and thereby the total amount of encapsulated microbeads can be scaled as necessary.

Step-by-step instructions for the preparation of FluoSurf-based emulsions

- Ready-made 2% FluoSurf oil-surfactant mixture can be obtained commercially
- Separate 10^7 microbeads from liquid using a magnetic rack for paramagnetic microbeads or via centrifugation for non-paramagnetic microbeads.
- Resuspend microbeads in 200 µl PURExpress Protein Synthesis reagent (prepared according to manufacturer's instructions).
- Keep microbead suspension on ice.
- Transfer 600 µl 2% FluoSurf oil-surfactant mixture to a 15 ml conical tube.
- Vortex the oil-surfactant mixture without a lid. For FluoSurf-based emulsions strong vortexing is necessary. The introduction of air is of no concern for FluoSurf-based emulsions. In our case, the vortexer VV3 by VWR was used on the setting 5 of 6 (2750 RPM) with a 5 mm orbital diameter.
- Sonicate the aqueous microbead suspension briefly for 5 seconds in a sonicator bath
- Aspirate the microbead suspension into a pipette tip-derived nozzle.
- Slowly (over the course of 10 s) add the microbead suspension to the vortexing oil surfactant mixture. Keep the pipette tip orifice close to the wall of the conical tube during the addition (see Supplementary Video 3).
- After addition, continue vortexing for **5 more minutes** to finalize the emulsion (final droplet volumes can be further reduced by increasing the vortex time).

- Check the emulsion via microscopy. Transfer 8 µl of the emulsion on a microscopy slide and place a microscopy cover on top. Best practice: Evaluate the monoclonality of the microbeads in emulsion droplets. When overcrowding with microbeads is a persistent issue, increase the volume of the aqueous reagent or reduce the amount of microbeads. Furthermore, evaluate droplet diameters microscopically. Droplet diameters can be further reduced by increasing the additional vortex time after addition of the aqueous phase.
- The total emulsion volume and thereby the total amount of encapsulated microbeads can be scaled as necessary.

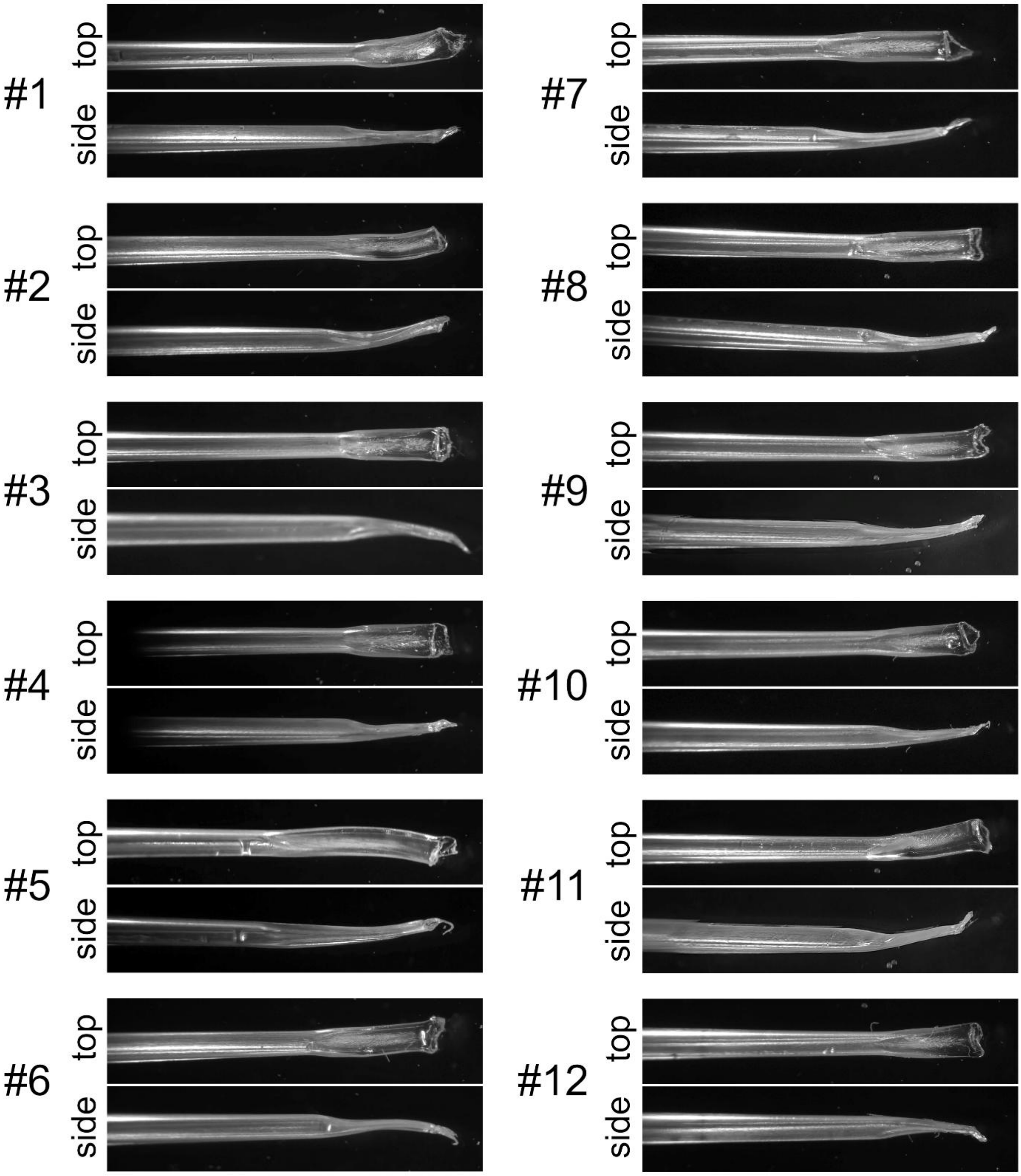

Supplementary Figure 1. Gallery of pipette tip-derived nozzle morphologies. For reference, 29 out of 34 production attempts were successful. Twelve nozzles were microscopically evaluated in more detail. Dimensional measurements are shown in Supplementary Table 1. In general, the morphology variation of the pipette tip-derived nozzles was quite diverse, however differences in nozzle morphology did not noticeably affect their functionality. Properly restricted liquid flow was the important factor determining success.

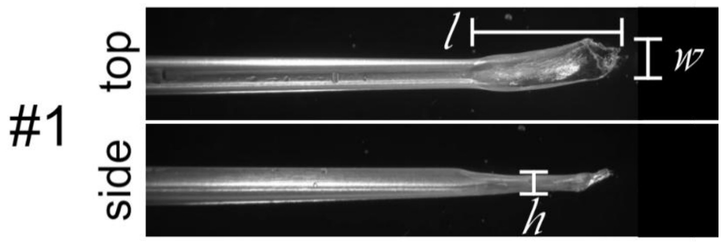

Nozzle ID	Length (l)	Width (w)	Height (h)
1	3.69	1.0	0.22
2	3.25	0.91	0.35
3	3.02	0.92	0.21
4	2.82	0.93	0.22
5	4.3	0.80	0.28
6	3.35	0.84	0.26
7	4.00	0.87	0.28
8	3.27	0.80	0.27
9	3.81	0.94	0.27
10	3.12	1.02	0.24
11	3.60	1.05	0.24
12	2.79	1.07	0.22

Supplementary Figure 2. Dimensional measurements were obtained for twelve nozzles fabricated from pipette tips. Measurements were conducted using a caliper at the specific locations indicated in the illustration above. Dimenstions are given in millimeter (mm).

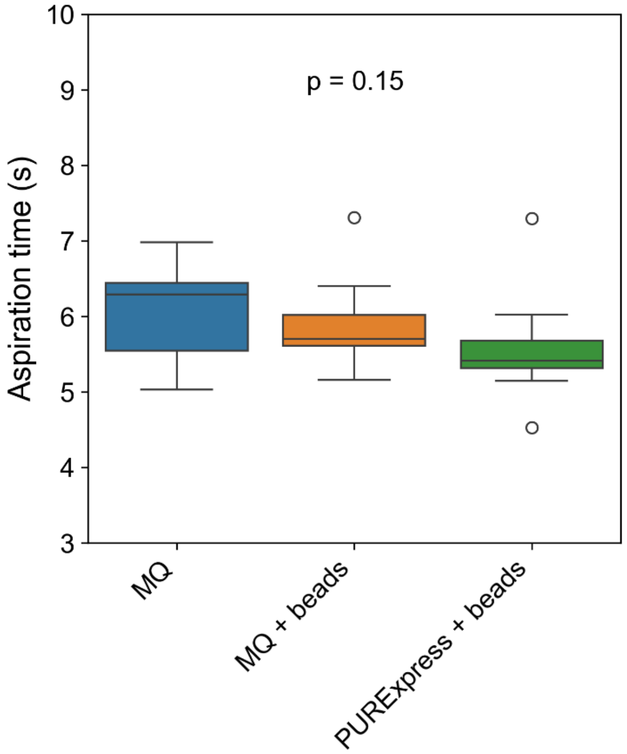

Nozzle ID	MQ H_2O	MQ H_2O + beads	PURExpress + beads
1	5.58 ± 0.90	5.71 ± 0.86	6.02 ± 0.15
2	5.43 ± 0.28	5.46 ± 0.78	5.33 ± 0.13
3	6.95 ± 0.91	7.31 ± 0.50	7.29 ± 1.50
4	5.03 ± 0.11	5.16 ± 0.43	4.52 ± 0.36
5	6.49 ± 1.48	5.96 ± 0.36	5.28 ± 0.50
6	6.32 ± 0.40	6.20 ± 0.15	5.15 ± 0.51
7	5.21 ± 0.52	5.65 ± 0.84	5.85 ± 1.23
8	6.98 ± 0.51	5.84 ± 0.32	5.43 ± 0.17
9	6.42 ± 0.89	5.68 ± 0.10	5.54 ± 0.18
10	6.26 ± 0.40	5.70 ± 0.23	5.39 ± 0.45
11	6.43 ± 0.34	6.40 ± 0.42	5.33 ± 0.26
12	5.74 ± 0.41	5.49 ± 0.22	5.62 ± 0.68

Supplementary Figure 3. Aspiration times of twelve pipette tip-derived nozzles using various liquid compositions. Top, box plot illustrating the influence of liquid composition and the presence of microbeads on the aspiration times of fabricated pipette tip-derived nozzles. Three conditions were tested: 200 µL of Milli-Q (MQ) water without microbeads, MQ water containing 10^7 microbeads, and PURExpress containing 10^7 microbeads. Aspiration times were measured and compared across these conditions. The table below reports the aspiration time for each nozzle and liquid composition as the mean of three replicate measurements (n = 3), with uncertainties indicated as ± values. No significant differences in aspiration times were observed among the tested liquid compositions. Statistical analysis was performed using one-way ANOVA.

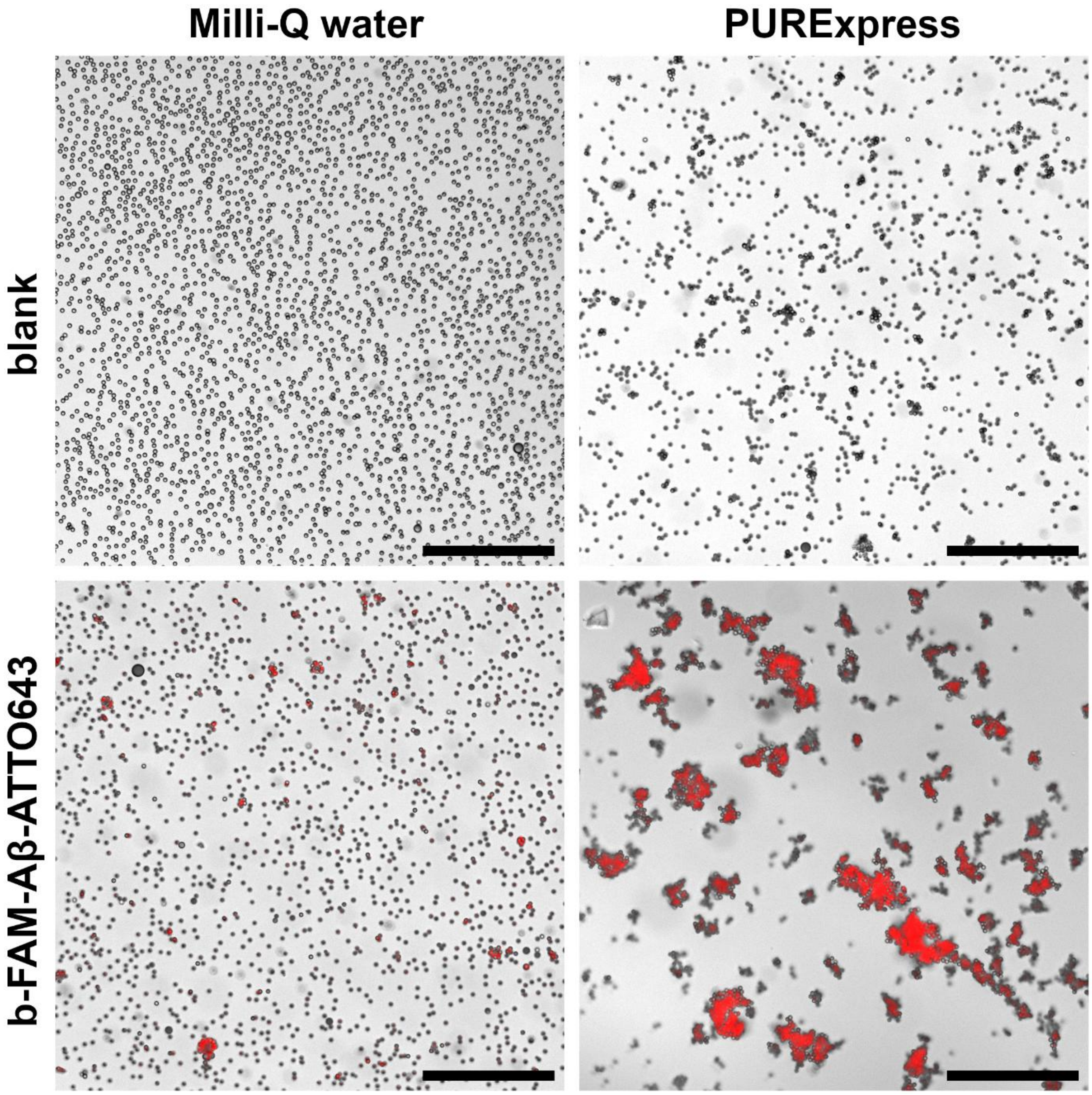

Supplementary Figure 4. Buffer composition and surface-modification influences aggregation propensity of paramagnetic microbeads. Overlay of brightfield and ATTO643 fluorescence channel micrographs of undecorated (blank) and peptide-decorated microbeads (10^6 peptides b-FAM-Aβ-ATTO643 per microbead) in pure Milli-Q water and PURExpress cell-free protein expression reagent. Upper left, undecorated (blank) microbeads in pure Milli-Q water were uniformly dispersed without observable aggregation. Upper right, blank microbeads in PURExpress reagent. Microbeads tended to form smaller clusters. Lower left, peptide decorated microbeads in pure Milli-Q water. Microbeads tended to form smaller clusters. Lower right, peptide decorated microbeads in PURExpress reagent. Microbeads severely aggregated with clusters reaching sizes of up to 100 µm. Scalebars, 100 µm.

a)

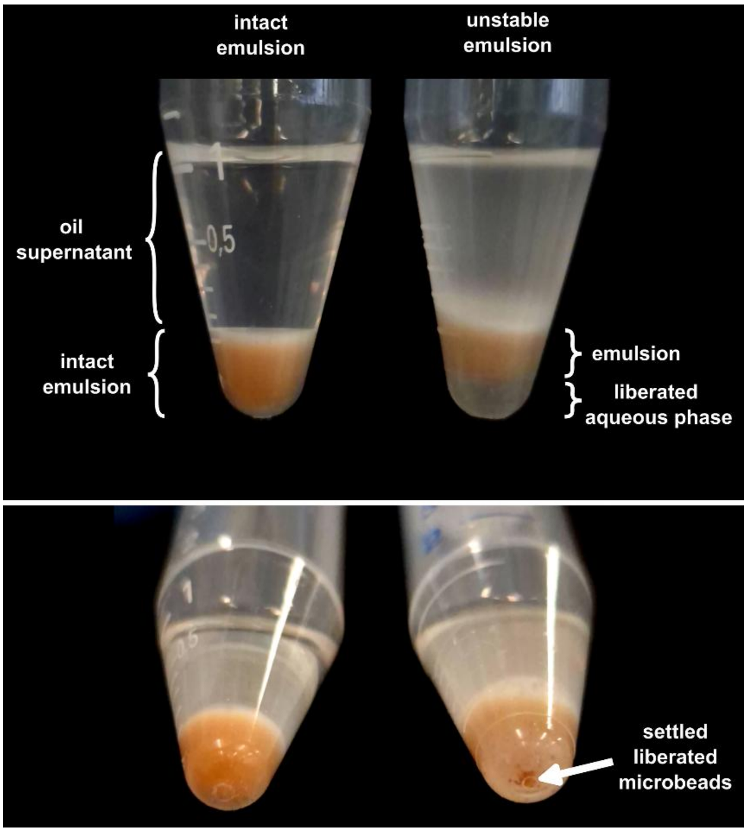

b)

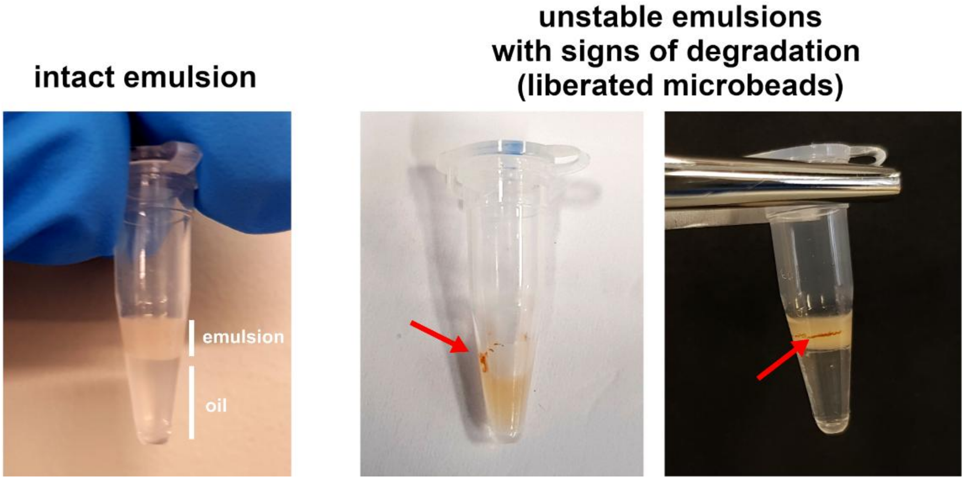

Supplementary Figure 5. Visual signs of emulsion degradation. a) Abil-EM 90-based emulsions. Left, a stable emulsion after 1 week of incubation at RT. Right, an unstable emulsion after 1 week of incubation. At the bottom of the tube liberated aqueous liquid and settled liberated microbeads can be seen. In case of severely unstable emulsions, this effect can be seen a few minutes to hours after emulsification. b) FluoSurf-based emulsions. Left, intact emulsion. In FluoSurf-derived emulsions, the emulsion layer floats on top of the oil phase. Middle, unstable emulsion. Microbeads can be seen clumped at the side of the tube (red arrow). Right, another unstable emulsion with microbeads sticking to the tube wall (red arrow).

Abil EM 90 FluoSurf

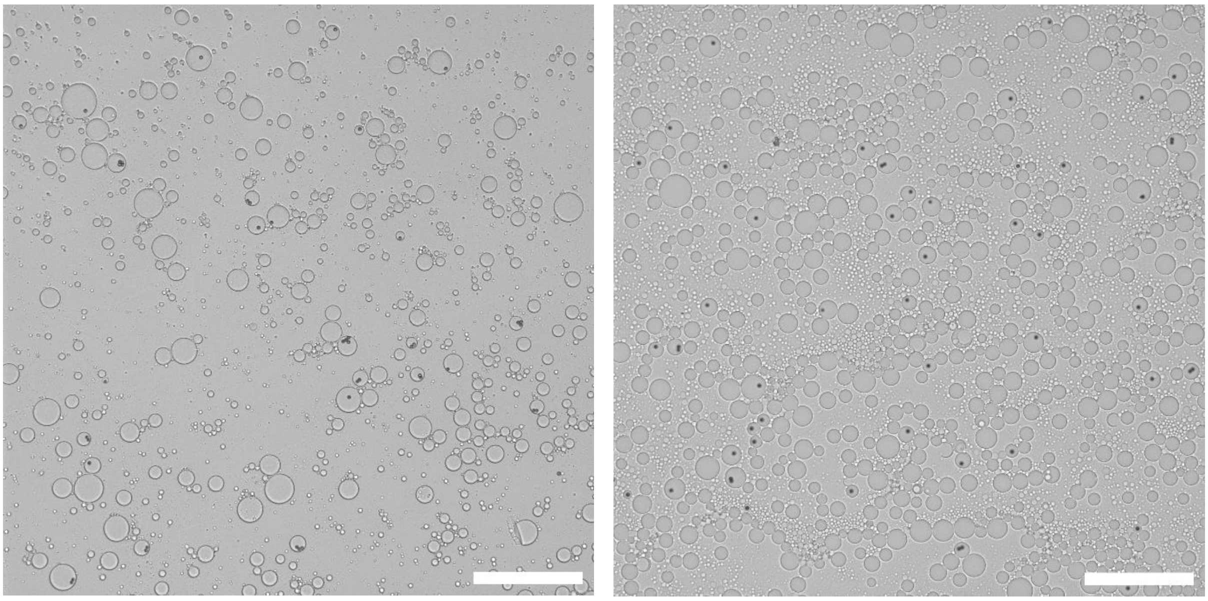

Supplementary Figure 6. Stable emulsions prepared with pipette tip-derived nozzles after more than 2 month (85 days) of incubation. Both Abil EM 90 and FluoSurf-based emulsions remained stable. Scalebar 100 μm.

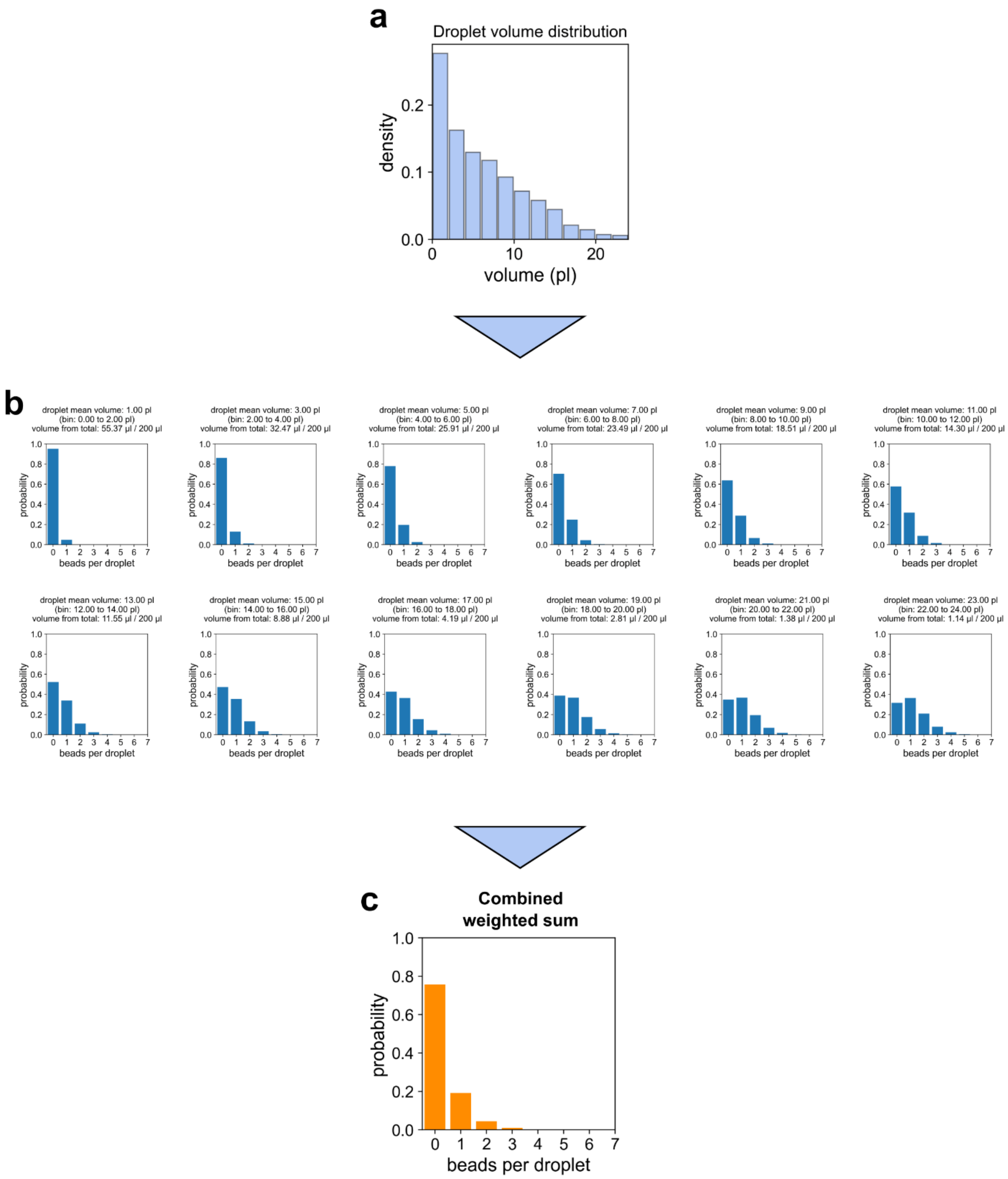

Supplementary Figure 7. Calculation of the expected microbead Poisson distribution based on the droplet volume distribution of FluoSurf-based emulsions. a) Droplet volume distribution, determined from N=3 emulsions with a total of 4874 analyzed droplets. b) The distribution was divided into 2 pL sized bins, and Poisson distributions were calculated for each fraction based on their respective share of the total aqueous reagent volume. c) A weighted sum of these distributions was used to estimate the expected microbead distribution across the entire emulsion.
